# An *FTO* Gene Variant Moderates the Association between Parental Restriction and Child BMI

**DOI:** 10.1371/journal.pone.0155521

**Published:** 2016-05-19

**Authors:** Alison Tovar, Jennifer A. Emond, Erin Hennessy, Diane Gilbert-Diamond

**Affiliations:** 1 Department of Nutrition and Food Sciences, University of Rhode Island, Kingston, RI, 02881, United States of America; 2 Department of Epidemiology, Geisel School of Medicine at Dartmouth College, Lebanon, NH, 3756, United States of America; 3 Friedman School of Nutrition Science and Policy, Tufts University, Boston, MA, 02111, United States of America; Laval University, CANADA

## Abstract

**Objective:**

This study aimed to explore whether a common variant in the *FTO* gene moderates the relationship between parental restriction and child BMI.

**Methods:**

This study reports on baseline data from 178 parent-child (ages 9–10 years) dyads. Parents completed the Child Feeding Questionnaire and reported on socio-demographic characteristics. Each child’s height, weight and *FTO* rs9939609 genotype was assessed. Ordinary least squares regression was used to fit the child’s BMI-percentile on parental restriction and the child’s *FTO* genotype, adjusted for covariates. A likelihood ratio test was used to compare a model with and without a multiplicative interaction term between restriction and genotype.

**Results:**

Most participants (93.3%) were white, non-Hispanic. Twenty-three percent of children were overweight/obese and *FTO* genotype was associated with weight status. Mean parental restriction was statistically higher among overweight/obese vs. normal weight children: 3.3 (SD 0.8) vs. 2.8 (SD 1.0); t-test p-value = 0.002. Parental restriction was positively associated with child BMI-percentile and BMI-z only among children with two copies of the high-risk *FTO* allele (p for interaction = 0.02), where each one-point increase in parental restriction was associated with a 14.7 increase in the child’s BMI-percentile or a 0.56-point increase in the child’s BMI z-score.

**Conclusion:**

For only the children with two high-risk alleles, parental restriction was positively associated with child BMI-percentile.

## Introduction

Childhood obesity is complex and results from both genetic and environmental factors [1]. One genetic factor that has been associated with child obesity is the *FTO* gene [2–5]. Variations in that gene have been tied to differences in body mass index and a risk of obesity [6–8]. Approximately 16% of the general population is homozygous for the common *FTO* rs9939609 risk variant (AA), and the odds of obesity among this group are approximately 70% higher for adults and children >7 years old compared to those who are homozygous for the wild type variant (TT) [9]. The association appears additive, as heterozygotes have an approximately 35% increased odds compared to those who are homozygous wild type (TT) [9]. Although the precise mechanism of *FTO*’s action is not known, its mRNA is highly expressed in the hypothalamus, a site for regulation of energy balance and appetite. This *FTO* risk allele may contribute to obesity by decreasing satiety responsiveness [10].

Environmental factors also play an important role in contributing to the rise in the prevalence of obesity which has occurred over the past 30 years [11]. Specifically, Western environments have become “obesogenic”, meaning that the environment is replete with easy access to energy-dense, nutrient poor foods and cues to consume such foods [12]. Additionally, parental feeding practices have come under scrutiny for their potential to disrupt children’s ability to self-regulate intake and increase the risk of obesity. In particular, parental restriction or controlling children’s access to and intake of certain foods is associated with child overeating and obesity [13–15]. Restrictive practices can be described as parents’ deliberate limiting on the amount and type of food, typically access to energy dense foods. Although caregivers may think this is a strategy that might promote a healthy diet and weight, restricting access to certain foods may have the opposite effect whereby children increase their preference for those foods [16]. One longitudinal study found that greater parental restriction at age 5 was associated with greater increases in BMI z-scores by age 7 only among mothers who were overweight prepregnancy [17], suggesting that restrictive feeding practices by parents may interact with child’s genetic predisposition for obesity to promote excess weight. Given that the development of childhood obesity is multifaceted and complex; hypothesized to be driven by gene-environment interactions, it is possible that depending on a child’s higher risk genetic predisposition, the influence of parental restriction on child BMI may differ. However, few studies have explored this interaction between a child’s genotype and feeding practices. This study aims to fill this gap and assess whether a child’s *FTO* genotype moderates the relationships between parental restriction and child BMI. We hypothesize that because children with one high risk allele report more frequent loss of control in eating, they may be more likely to engage in unhealthy eating behaviors in response to parental restriction which in turn influences their risk for obesity.

## Methods

### Design and Study Participants

Participants were recruited between August 2013-March 2015 for an experimental study to examine associations between food advertising exposure and eating behaviors among pre-adolescents. Full details of the study can be found elsewhere (Gilbert-Diamond, in-press), but are summarized here. Briefly, children aged 9–10 years and one of their parents (or guardians) were recruited from the community using a pediatric clinic contact list and community fliers, and dyads participated in the study by going to a college lab. Once informed consents were signed, children were asked to eat a standardized lunch and then viewed an age-appropriate, 34-minute TV program. Immediately afterward children completed study questionnaires described below. Parents also completed a study questionnaire while their child completed the study assessments (S1 Table). Eligibility criteria included English fluency, absence of food allergies and dietary restrictions, absence of health conditions or medication use that may impact appetite, and willingness to participate in a 2-hour study. Of the 252 participants screened, 224 were eligible and 200 enrolled. There were 21 families that enrolled two children into the study, and we limited analyses to unrelated participants (n = 179) by randomly selecting one child in each sibling pair. Analyses further excluded one underweight child (see below). Final analyses therefore included 178 children and one of their parents. Participating children provided assent for study procedures, and parents provided consent for their children and themselves. The Committee for the Protection of Human Subjects at Dartmouth College approved of all study procedures.

### Parental Restriction

Parents completed a modified version of the parental restriction subscale of the Child Feeding Questionnaire [18]. The original, 8-item restriction scale assesses parental feeding practices as related to keeping track of a child’s intake of highly-palatable foods (e.g., I have to be sure that my child does not eat too many sweets), limiting access to such foods (e.g., I intentionally keep some foods out of my child's reach) and using food as a reward (e.g., I offer my child her favorite foods in exchange for good behavior). Items responses are on a 5-point Likert scale, anchored at 1 (disagree) and 5 (agree). The final score is the average over all 8 items with higher scores indicating greater restriction. The parental restriction subscale has appropriate internal consistency (Cronbach’s alpha = 0.73) [18], and scores have been positively associated with increased child weight in several studies [19]. The parental restriction scale used in this study was slightly modified from the original, in that two separate items assessing food as a reward (i.e., I offer sweets to my child as a reward for good behaviors; I offer my child her favorite foods in exchange for good behavior) were combined into one item (i.e., I offer my child her favorite foods or sweets in exchange for good behavior). Thus, parental restriction in this analysis was a 7-item scale. Internal consistency remained appropriate (Cronbach’s alpha = 0.79), and the final score was a mean over the 7 items (range 1–5).

### FTO rs9939609 genotyping

Detailed genotyping methods have been previously described (Gilbert-Diamond, in-press). In brief, DNA was isolated from buccal cells collected at the beginning of the experiment using swabs (Isohelix, Kent, U.K.). Genotyping for rs9939609 was then done with real time PCR and Taqman chemistry. All 200 samples were successfully genotyped for rs9939609 and there was 100% genotyping consistency among the 10% of samples that were blindly replicated.

### Additional measures

Parents reported on their child’s age, gender, race, and ethnicity. Parents reported on their relationship to the child, marital status, household income and educational attainment for themselves and their spouse (if appropriate). For analyses, the educational attainment of the mother was used because the vast majority of parents who participated in the study were mothers and mothers continue to play a primary role in child feeding [20,21]. The child’s weight and height were measured at the study visit using a Seca 763 Medical Scale and Seca 213 Stadiometer (Hamburg, Germany) respectively. All measurements were taken without shoes and in light indoor clothing. Measurements were used to compute age- and sex-adjusted BMI-percentiles and age- and sex-adjusted BMI z-scores using the Center for Disease Control and Prevention 2000 growth charts [22]. Overweight or obese was defined as ≥85^th^ percentile. One participant was classified as underweight per the Centers for Disease Control and Prevention criteria (<5^th^ BMI-percentile) [23], and all analyses excluded this one participant.

### Analyses

We completed all analyses using the child’s BMI-percentile and BMI z-score as the outcome. We focus on the child’s BMI-percentile as the primary outcome given that it is more commonly used in clinical settings. Distributions of child’s BMI-percentiles and BMI z-scores were compared across child, parent and household characteristics using unadjusted Student’s t-tests or ANOVAs. Similarly, the distributions for parental restriction as well as the child’s genotype across child, parent and household characteristics were compared. Unadjusted correlations between parental restriction and the child’s BMI-percentile and BMI z-score were computed using Pearson’s correlations. Scatterplots of parental restriction against the child’s BMI-percentile and BMI z-score stratified by *FTO* genotype were visually inspected, and those plots supported linear associations. Ordinary least squares regression was used to fit the child’s BMI-percentile and BMI z-score on parental restriction and the child’s *FTO* genotype, adjusted for the child’s gender, the mother’s educational attainment and annual household income. Those covariates were included because they were associated with the child’s BMI-percentile and BMI z-score or parental restriction at the p<0.10 level in unadjusted bivariate analyses. To assess if the relation between parental restriction and the child’s BMI-percentile and BMI z-score differed based on the child’s *FTO* genotype, an interaction term between parental restriction and the child’s *FTO* genotype in the regression model was included. A likelihood ratio test was used to compare those two nested models and p<0.10 was considered evidence of significant effect modification. Adjusted point estimates and 95% confidence intervals for stratum-specific associations [24] were presented. To improve the interpretation of results from that model, we also presented mean child BMI percentile by parental restriction, dichotomized at the median, stratified by *FTO* genotype. All analyses were completed using the R language and environment for statistical computing, version 3.0.2 [25].

## Results

Analyses included data from 178 normal, overweight or obese children and their parent (or guardian). Children’s age and gender were equally distributed among the sample. Most participants (93.3%) were white, non-Hispanic. Mean BMI-percentile was 60.1 (standard deviation, SD = 26.8), mean BMI z-score was 0.39 (SD = 0.95), and 41 children (23.0%) were overweight or obese.

### Bivariate associations: child BMI, demographic and household characteristics

Table 1 presents the distribution of child BMI-percentile and BMI z-score across child, parent and household characteristics and child’s *FTO* genotype. A greater BMI-percentile and z-score was statistically associated with male gender and lower levels of education attainment for the mother. Mean BMI-percentiles and z-scores were also statistically different by the child’s *FTO* genotype. Among children with no copies of the high-risk allele (i.e., TT), mean BMI-percentile was 60.7, BMI z-score was 0.36 and 17.7% of children were overweight or obese. In comparison, among children with two copies of the high-risk allele (i.e., AA), mean BMI-percentile was 73.1, BMI z-score 0.93 and 41.4% of children were overweight or obese. The child’s *FTO* genotype was not associated with any child, parent or household characteristics (data not shown) other than the child’s BMI-percentile, BMI z-score or weight status.

**Table 1:** Bivariate associations of the child’s BMI-percentile and BMI z-score with child, parent household characteristics and child’s *FTO* genotype.

	Overall	BMI-Percentile		BMI z-score	
	N	Mean (SD)	p-value^1^	Mean (SD)	p-value^1^
Overall	178	60.1 (26.8)	--	0.39 (0.95)	--
**Child characteristics**					
Age, years					
9	97	63.1 (25.5)	0.11	0.50 (0.92)	0.10
10	81	56.6 (28.1)		0.26 (0.98)	
Sex					
Female	91	54.0 (25.1)	<0.01	0.18 (0.86)	<0.01
Male	87	66.6 (27.1)		0.61 (1.00)	
Race					
White only	166	60.0 (26.2)	0.53	0.38 (0.93)	0.65
African-American	5	48.4 (39.4)		0.12 (1.61)	
Asian-American	3	72.9 (34.9)		0.86 (1.15)	
Other	4	70.4 (34.0)		0.73 (1.11)	
Ethnicity					
Hispanic or Latino	6	66.1 (30.1)	0.58	0.55 (0.98)	0.68
Non-Hispanic or Latino	172	59.9 (26.8)		0.38 (0.95)	
**Parent characteristics**					
Relationship to child					
Mother	148	60.5 (26.6)	0.30	0.41 (0.94)	0.28
Father	28	56.2 (28.1)		0.22 (0.99)	
Other	2	85.4 (15.9)		1.24 (0.83)	
Mother's education level					
Associate’s degree or less	36	69.7 (29.6)	0.01	0.79 (1.08)	<0.01
Bachelor’s degree	52	62.5 (25.1)		0.47 (0.90)	
Graduate/professional degree	90	54.9 (25.6)		0.18 (0.88)	
Marital status					
Married	150	59.8 (26.9)	0.95	0.38 (0.95)	0.94
Single, never married	8	62.9 (31.1)		0.51 (1.05)	
Separated or divorced	17	60.2 (26.1)		0.38 (0.98)	
Other	3	67.6 (27.3)		0.66 (1.04)	
**Household characteristics**					
Annual household income					
<$65,000	44	63.3 (27.5)	0.24	0.51 (1.00)	0.12
$65,000–$144,999	83	62.1 (27.7)		0.48 (1.02)	
$145,000–$225,000	32	56.4 (26.4)		0.21 (0.83)	
>$225,000	19	50.3 (19.8)		0.00 (0.56)	
**Child’s *FTO* genotype**					
TT	62	60.7 (24.9)	<0.01	0.36 (0.82)	<0.01
AT	87	55.4 (27.6)		0.23 (0.96)	
AA	29	73.1 (24.6)		0.93 (1.02)	

^1^P-values are from t-tests or ANOVAs to compare mean BMI-percentile or BMI z-score by category levels.

### Bivariate associations: child BMI and parental restriction

Mean parental restriction was 2.9 (SD = 0.9), and parental restriction was not statistically associated with any child, parent or household characteristics, except for the child’s weight status. Specifically, mean parental restriction was statistically lower among normal weight children as compared to overweight or obese children: 2.8 (SD = 1.0) vs. 3.3 (SD = 0.8); t-test p-value = 0.002. The correlations between parental restriction and the child’s BMI-percentile and BMI z-score were positive and statistically significant, yet low (Pearson’s rho 0.17; p = 0.023, Pearson’s rho 0.20; p<0.01, respectively). However, the magnitudes of those correlations differed by the child’s *FTO* genotype (Table 2) and were high (Pearson’s rho = 0.60; p<0.001, Pearson’s rho = 0.56; p<0.01, respectively) among participants with two copies of the high-risk allele. The correlations between parental restriction and child’s BMI percentile or z-score were similar. Specifically, increased parental restriction was positively associated with the child’s BMI-percentile and z-score only among children with two copies of the high-risk allele. Mean parental restriction did not differ by the child's genotype (p = 0.83)

**Table 2:** Correlation between parental restriction and the child's BMI-percentile overall and stratified by the child's *FTO* genotype^1^.

	N	Pearson's Rho	p-value
Overall	178	0.17	0.02
By the child’s *FTO* genotype			
TT	62	0.18	0.16
AT	87	0.05	0.66
AA	29	0.60	<0.001

^1^Among 178 normal, overweight or obese children enrolled in a media study.

### Linear regression models: child BMI on parental restriction and child’s *FTO* genotype

In an adjusted linear regression model (Table 3), parental restriction and the child’s *FTO* genotype each remained statistically associated with the child’s BMI-percentile and BMI z-score. Specifically, each one-unit increase in parental restriction was significantly associated with a 4.5-point increase in the child’s BMI-percentile. Additionally, children who had two copies of the high-risk allele had a mean BMI-percentile that was 12.9 points greater than the mean of children who had zero copies of the high-risk allele. Participants with one copy of the high-risk allele had a lower mean BMI percentile as compared participants with no copies, (difference in mean BMI-percentile (standard error of the mean): -8.2 (4.1); p = 0.05. Findings from the model that fit child BMI z-score as the outcome were similar (Table 3).

**Table 3:** Adjusted associations between child, parent and household characteristics with the child’s BMI-percentile or BMI z-score^1^^,^^2^.

	Outcome			
	BMI-percentile		BMI z-score	
	$\hat{\beta }$ (SE)	p-value	$\hat{\beta }$ (SE)	p-value
Sex				
Female	Reference	--	Reference	--
Male	14.0 (3.7)	<0.001	0.47 (0.13)	<0.001
Mother's education level				
Associate’s degree or less	Reference	--	Reference	--
Bachelor’s degree	-9.4 (5.5)	0.09	-0.42 (0.19)	0.03
Graduate or professional degree	-15.8 (5.3)	<0.01	-0.65 (0.18)	<0.001
Annual household income				
<$65,000	Reference	--	Reference	--
$65,000–$144,999	0.0 (4.8)	0.99	0.05 (0.17)	0.76
$145,000–$225,000	-2.4 (6.1)	0.70	-0.10 (0.21)	0.65
>$225,000	-6.5 (7.2)	0.37	-0.21 (0.25)	0.40
CFQ: Restriction	4.5 (2.0)	0.02	0.19 (0.07)	<0.01
*FTO* genotype				
TT	Reference	--	Reference	--
AT	-8.2 (4.1)	0.05	-0.24 (0.14)	0.09
AA	12.9 (5.6)	0.02	0.59 (0.19)	<0.01

$\hat{\beta }$ (SE): beta coefficient and standard error

^1^Among 178 normal, overweight or obese children enrolled in a media study.

^2^Ordinary least squares regression used. All model covariates presented

**Fig 1:**
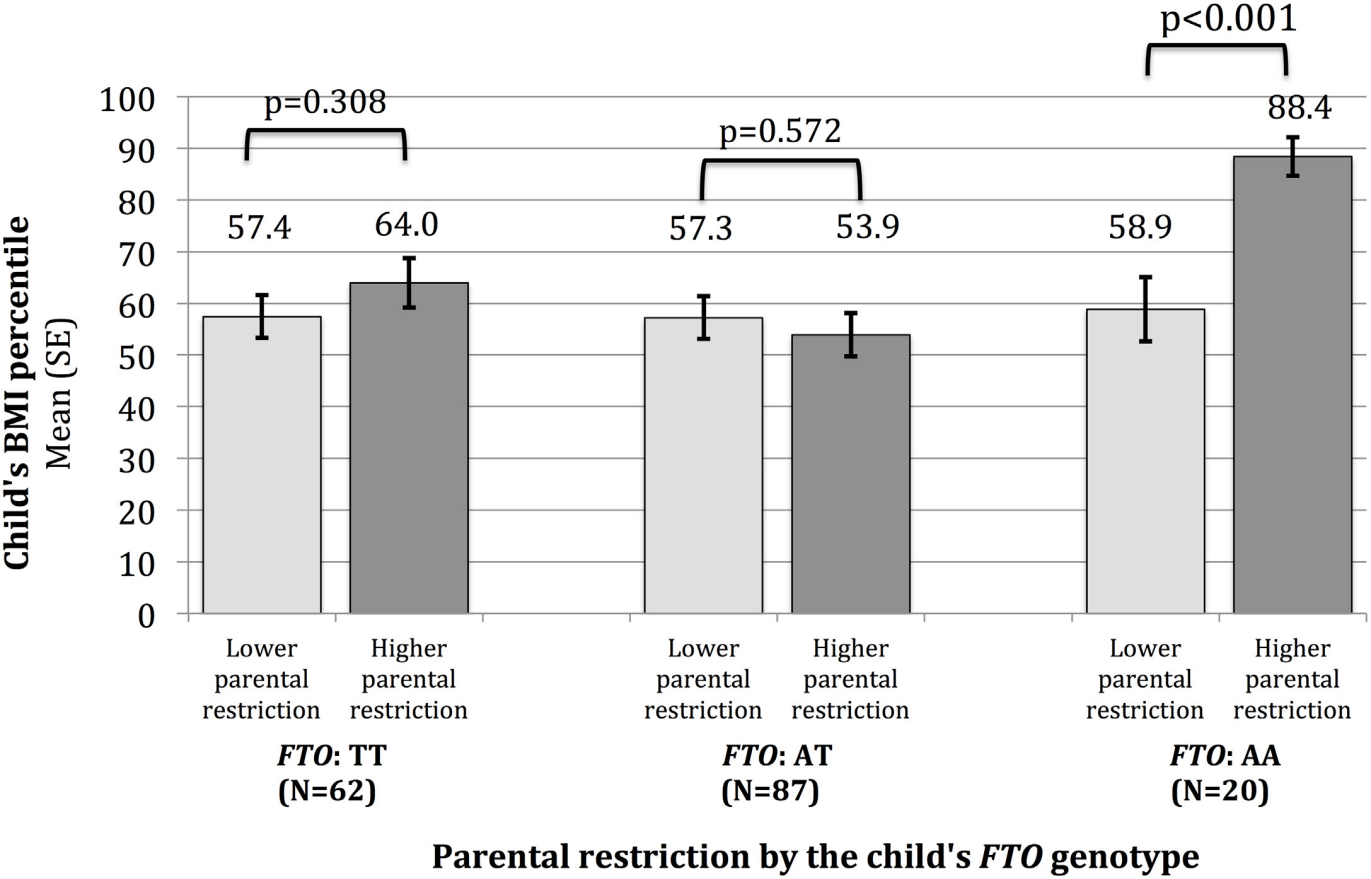
Mean child BMI-percentile by parental restriction, stratified by the child’s *FTO* genotype. Among 178 normal, overweight or obese children enrolled in a media study. Parental restriction dichotomized at the median value (1.0–2.8 vs. 2.9–5.0). Mean scores compared by median using Student’s t-tests.

There was a statistically significant interaction between parental restriction and the child’s *FTO* genotype on the child’s BMI-percentile (p = 0.02) and BMI z-score (p = 0.02). Specifically, parental restriction appeared positively associated with the child’s BMI-percentile and z-score only among children with two copies of the high-risk allele. Each one-point increase in parental restriction was associated with a 14.7-point increase in the child’s BMI-percentile or a 0.56 point increase in the child’s BMI z-score. To help illustrate that finding, we compared the child’s BMI-percentile across parental restriction dichotomized at the median, stratified by the child’s *FTO* genotype (Fig 1). As demonstrated in Fig 1, mean child BMI-percentile was statistically greater in the upper vs. lower median of parental restriction score for children with an AA genotype only (88.4 vs. 58.9 percentile; p<0.001). Findings were consistent when using BMI z-score as the outcome.

## Discussion

In this study of 178 children aged 9–10 years old, we found that parental restriction and *FTO* genotype were both independently associated with increased child BMI, adjusted for child’s gender, mother’s education level and household income. Further, the positive association between parental restriction and child BMI appeared limited to children who had two copies of the *FTO* high-risk allele. Our findings add to the growing literature which support that the development of childhood obesity is multifaceted and complex; hypothesized to be driven by gene-environment interactions [10]. Findings further suggest that interventions to prevent childhood obesity may be most effective when tailored to children’s genetic predisposition.

Similar to other studies we found that children with two copies of the *FTO* high-risk allele (i.e., AA) were more likely to be overweight/obese (41%) compared to those with no copies (i.e., TT, with 18% overweight or obese) [7,26,27]. Unlike other studies, we did not observe an additive effect of the risk alleles on BMI, which may be related to our relatively small sample size. Our findings that greater parental restriction related to increased child weight status and BMI are in line with what others have found in that mean parental restriction was greater among overweight/obese children as compared to normal weight children [17]. Restrictive feeding is usually coercive, intrusive and parent-centered [28]. Restricting food intake has been associated with increased children’s intake of and attraction to the restricted foods, usually energy-dense palatable foods, therefore thought to promote long-term dysregulation in eating and obesity [16,29,30].

The field of parental feeding is slowly detangling the complexities of having different feeding strategies and discovering that a “one-size” approach may not fit all. Environmental and biologic factors come into play as well as the nature of the direction that parents provide their children. Our finding of an interaction between feeding practices and genotype supports the complex nature of these relationships. Our findings are also consistent with those of Faith et al. whereby among children with heavier mothers, parental restriction when children were age five years predicted higher BMI-z scores at age seven years, even after controlling for child weight status at age three [17]. Our findings further extend previous work by identifying a specific gene, *FTO*, which has been associated with obesity but has not previously been explored with feeding practices. The *FTO* rs9939609 risk allele may have a role in the control of food intake and, particularly in decreasing satiety responsiveness. Wardle et al. found that homozygote high-risk children had significantly reduced satiety responsiveness scores and also found that the association of the high-risk genotype with increased adiposity was explained in part through effects on satiety responsiveness [31]. Similarly, children with at least one high risk allele reported more frequent loss of control in eating [32]. We hypothesize that children with the high-risk *FTO* rs9939609 genotype may be more likely to engage in unhealthy eating behaviors in response to parental restriction. It is possible that when children have an underlying difficulty in regulating food intake, parental restriction leads to a greater preference for and intake of restricted foods. They may also have trouble developing healthy eating practices as they are experiencing discordant messages whereby their biology is prompting them to eat and their parents are expecting them to practice self-restraint. In a previous analysis, we found that parental restriction was associated with eating in the absence of hunger (EAH) (Gilbert-Diamond, *in press*), but the association did not vary by the *FTO* rs9939609 genotype. Future research in this area is needed to better understand the mechanism by which this *FTO* genotype moderates the association between restrictive feeding and child weight.

While restriction has been associated with obesity and increases in unhealthy food consumption, the direction of this relationship remains unclear. It is possible that the directionality of the relationship could in fact be reversed. For example, it is possible that parents who are concerned about their child’s weight use more restrictive practices as a result, and/or that parents use restrictive feeding practices in response to a child’s tendency to overeat [33]. Some longitudinal studies have found this to be true in that parents were more likely to use restriction in response to their child’s weight gain in early childhood [7,34]. It may be that parents are accurately perceiving their child's biologically driven poor satiety responsiveness, and that they correctly realize that if they do not provide external cues to stop eating, the child will become even more obese because the child's own internal cues to stop eating are not reliable (perhaps due to the FTO risk alleles, or some other biological mechanism). Given the study’s cross-sectional design, we are unable to disentangle the directionality of our findings; however, our data can inform hypotheses of future studies.

This study begins to address a clear gap in the literature around gene-environment interactions; however, it does have certain limitations. First, we did not assess other candidate genes which have been associated with obesity such as melanocortin-4 receptor (MC4R) gene as well as *BDNF*[35]. We however chose the *FTO* gene given the strength of the evidence linking *FTO* rs9939609 to obesity in both children and adults and the large prevalence of individuals with risk alleles [36]. Second, we only assessed restriction as the sole feeding practice given the consistent findings of this practice with unhealthy dietary intake and obesity[37]. The growing literature however has begun to explore other autonomy supportive or structuring practices [38] that should be explored in the future. Third, there has been some controversy over the use of the restriction subscale of the CFQ and if the items appropriately load onto the construct of restriction [39–41]. However, this has been one of the most widely used feeding practice questionnaires that has shown strong criterion validity and makes understanding our findings in the context of the broader literature easier to directly compare. Finally our sample was primarily white middle-upper class with possible limited generalizability to other more diverse populations. Future research should explore gene-environment interactions in racially and ethnically diverse low-income populations where disparities of childhood obesity are evident.

In conclusion we found that parental restriction was positively associated with child BMI, but only for those who have two copies of the high-risk allele. Future research is needed to continue to understand the complexity of genetic and environmental feeding associations and to understand possible mechanisms. It is possible that because children with one high risk allele report more frequent loss of control in eating [32], they may be more likely to engage in unhealthy eating behaviors in response to parental restriction which in turn influences their risk for obesity. Given how hard it is to reach and work with parents around obesity prevention, it is possible that future obesity prevention interventions can be more effective if tailored for high-risk children. Our findings suggest that for these families emphasis on non-restrictive feeding practices may be more effective. Future work should also move towards exploring other aspects of positive feeding where parents may be more amenable to change.

## Supporting Information

S1 Table Parental Questionnaire. (PDF)

## References

[1] Bromer J (2001) Helpers, mothers, and preachers: the multiple roles and discourses of family child care providers in an African-American community. Early Childhood Research Quarterly 16: 313–327.

[2] Zhao J, Bradfield JP, Li M, Wang K, Zhang H, Kim CE, et al (2009) The role of obesity-associated loci identified in genome-wide association studies in the determination of pediatric BMI. Obesity (Silver Spring) 17: 2254–2257. 19478790 10.1038/oby.2009.159 PMC2860782

[3] den Hoed M, Ekelund U, Brage S, Grontved A, Zhao JH, Sharp SJ, et al (2010) Genetic susceptibility to obesity and related traits in childhood and adolescence: influence of loci identified by genome-wide association studies. Diabetes 59: 2980–2988. 10.2337/db10-0370 20724581 PMC2963559

[4] Bradfield JP, Taal HR, Timpson NJ, Scherag A, Lecoeur C, Warrington NM, et al (2012) A genome-wide association meta-analysis identifies new childhood obesity loci. Nat Genet 44: 526–531. 10.1038/ng.2247 22484627 PMC3370100

[5] Speliotes EK, Willer CJ, Berndt SI, Monda KL, Thorleifsson G, Jackson AU, et al (2010) Association analyses of 249,796 individuals reveal 18 new loci associated with body mass index. Nat Genet 42: 937–948. 10.1038/ng.686 20935630 PMC3014648

[6] Frayling TM, Ong K (2011) Piecing together the FTO jigsaw. Genome Biol 12: 104 10.1186/gb-2011-12-2-104 21349207 PMC3188788

[7] Frayling TM, Timpson NJ, Weedon MN, Zeggini E, Freathy RM, Lindgren CM, et al (2007) A common variant in the FTO gene is associated with body mass index and predisposes to childhood and adult obesity. Science 316: 889–894. 17434869 10.1126/science.1141634 PMC2646098

[8] Yang J, Loos RJ, Powell JE, Medland SE, Speliotes EK, Chasman DI, et al (2012) FTO genotype is associated with phenotypic variability of body mass index. Nature 490: 267–272. 10.1038/nature11401 22982992 PMC3564953

[9] Miller LS, Szych GA, Kantor SB, Bromer MQ, Knight LC, Maurer AH, et al (2002) Treatment of idiopathic gastroparesis with injection of botulinum toxin into the pyloric sphincter muscle. American Journal of Gastroenterology 97: 1653–1660. 12135014 10.1111/j.1572-0241.2002.05823.x

[10] Faith MS, Carnell S, Kral TV (2013) Genetics of food intake self-regulation in childhood: literature review and research opportunities. Hum Hered 75: 80–89. 10.1159/000353879 24081223

[11] Campbell MK (2015) Biological, environmental, and social influences on childhood obesity. Pediatr Res. 10.1038/pr.2015.208 26484623

[12] Swinburn B, Egger G, Raza F (1999) Dissecting obesogenic environments: the development and application of a framework for identifying and prioritizing environmental interventions for obesity. Prev Med 29: 563–570. 10600438 10.1006/pmed.1999.0585

[13] Faith MS, Scanlon KS, Birch LL, Francis LA, Sherry B (2004) Parent-child feeding strategies and their relationships to child eating and weight status. Obesity research 12: 1711–1722. 15601964 10.1038/oby.2004.212

[14] Ventura AK, Birch LL (2008) Does parenting affect children's eating and weight status? International Journal of Behavioral Nutrition and Physical Activity 5: 15 10.1186/1479-5868-5-15 18346282 PMC2276506

[15] Francis LA, Susman EJ (2009) Self-regulation and rapid weight gain in children from age 3 to 12 years. Archives of pediatrics & adolescent medicine 163: 297–302. 19349557 10.1001/archpediatrics.2008.579 PMC9433163

[16] Fisher JO, Birch LL (1999) Restricting access to palatable foods affects children's behavioral response, food selection, and intake. Am J Clin Nutr 69: 1264–1272. 10357749 10.1093/ajcn/69.6.1264

[17] Faith MS, Berkowitz RI, Stallings VA, Kerns J, Storey M, Stunkard AJ (2004) Parental feeding attitudes and styles and child body mass index: prospective analysis of a gene-environment interaction. Pediatrics 114: e429–436. 15466068 10.1542/peds.2003-1075-L

[18] Birch LL, Fisher JO, Grimm-Thomas K, Markey CN, Sawyer R, Johnson SL (2001) Confirmatory factor analysis of the Child Feeding Questionnaire: a measure of parental attitudes, beliefs and practices about child feeding and obesity proneness. Appetite 36: 201–210. 11358344 10.1006/appe.2001.0398

[19] Loth KA, MacLehose RF, Fulkerson JA, Crow S, Neumark-Sztainer D (2013) Food-related parenting practices and adolescent weight status: a population-based study. Pediatrics 131: e1443–1450. 10.1542/peds.2012-3073 23610202 PMC3639463

[20] Birch LL, Fisher JO (1998) Development of eating behaviors among children and adolescents. Pediatrics 101: 539–549. 12224660

[21] Savage JS, Fisher JO, Birch LL (2007) Parental influence on eating behavior: conception to adolescence. J Law Med Ethics 35: 22–34. 17341215 10.1111/j.1748-720X.2007.00111.x PMC2531152

[22] Kuczmarski RJ, Ogden CL, Guo SS, Grummer-Strawn LM, Flegal KM, Mei Z, et al (2002) 2000 CDC Growth Charts for the United States: methods and development. Vital Health Stat 11: 1–190. 12043359

[23] Meyers A, Joyce K, Coleman SM, Cook JT, Cutts D, Ettinger de Cuba S, et al (2013) Health of children classified as underweight by CDC reference but normal by WHO standard. Pediatrics 131: e1780–1787. 10.1542/peds.2012-2382 23690515

[24] Figueiras A, Domenech-Massons JM, Cadarso C (1998) Regression models: calculating the confidence interval of effects in the presence of interactions. Stat Med 17: 2099–2105. 9789916 10.1002/(sici)1097-0258(19980930)17:18<2099::aid-sim905>3.0.co;2-6

[25] R Core Team (2013). R: A language and environment for statistical computing. R Foundation for Statistical Computing V, Austria Available: http://www.R-project.org/.

[26] Larson N, Ward DS, Neelon SB, Story M (2011) What role can child-care settings play in obesity prevention? A review of the evidence and call for research efforts. J Am Diet Assoc 111: 1343–1362. 10.1016/j.jada.2011.06.007 21872698

[27] Wissow L, Gadomski A, Roter D, Larson S, Lewis B, Brown J (2011) Aspects of mental health communication skills training that predict parent and child outcomes in pediatric primary care. Patient Educ Couns 82: 226–232. 10.1016/j.pec.2010.03.019 20444568 PMC2947561

[28] Wissow LS, Gadomski A, Roter D, Larson S, Brown J, Zachary C, et al (2008) Improving child and parent mental health in primary care: a cluster-randomized trial of communication skills training. Pediatrics 121: 266–275. 10.1542/peds.2007-0418 18245417

[29] Tran L, Campbell L, Coletta D, Mandarino L, Katsanos C (2015) Skeletal Muscle beta-F1-ATPase Translation is Inhibited by Hyperlipidemia- Induced miR-127-5p Expression in Human Obesity. Faseb Journal 29.

[30] Jansen E, Mulkens S, Jansen A (2007) Do not eat the red food!: prohibition of snacks leads to their relatively higher consumption in children. Appetite 49: 572–577. 17490786 10.1016/j.appet.2007.03.229

[31] Shah P, Misra A, Gupta N, Hazra DK, Gupta R, Seth P, et al (2010) Improvement in nutrition-related knowledge and behaviour of urban Asian Indian school children: findings from the 'Medical education for children/Adolescents for Realistic prevention of obesity and diabetes and for healthy aGeing' (MARG) intervention study. Br J Nutr 104: 427–436. 10.1017/S0007114510000681 20370939

[32] Bromer MQ, Kantor SB, Wagner DA, Knight LC, Maurer AH, Parkman HP (2002) Simultaneous measurement of gastric emptying with a simple muffin meal using [13C]octanoate breath test and scintigraphy in normal subjects and patients with dyspeptic symptoms. Dig Dis Sci 47: 1657–1663. 12141833 10.1023/a:1015856211261

[33] Ishizaka K, Ishizaka T, Campbell DH (1959) Biologic activity of soluble antigen-antibody complexes. III. J Immunol 83: 105–115. 13853052

[34] Curkendall S, Patel V, Gleeson M, Campbell RS, Zagari M, Dubois R (2008) Compliance with biologic therapies for rheumatoid arthritis: do patient out-of-pocket payments matter? Arthritis Rheum 59: 1519–1526. 10.1002/art.24114 18821651

[35] Stefanini M, Campbell EW (1954) Studies on platelets. XII. Isolation and purification of the platelet thromboplastic factor; its physico-chemical and biologic properties in vitro and in vivo. Rev Hematol 9: 576–585. 14357983

[36] Karaman U, Campbell K, Frilot CF, Gomelsky A (2015) The Impact of Obesity on Outcomes after Retropubic Midurethral Sling for Female Stress Urinary Incontinence. Journal of Urology 193: E646–E646.

[37] Campbell JM, Lane M, Owens JA, Bakos HW (2015) Paternal obesity negatively affects male fertility and assisted reproduction outcomes: a systematic review and meta-analysis. Reprod Biomed Online 31: 593–604. 10.1016/j.rbmo.2015.07.012 26380863

[38] Vaughn AE, Tabak RG, Bryant MJ, Ward DS (2013) Measuring parent food practices: a systematic review of existing measures and examination of instruments. Int J Behav Nutr Phys Act 10: 61 10.1186/1479-5868-10-61 23688157 PMC3681578

[39] Boles RE, Nelson TD, Chamberlin LA, Valenzuela JM, Sherman SN, Johnson SL, et al (2010) Confirmatory factor analysis of the Child Feeding Questionnaire among low-income African American families of preschool children. Appetite 54: 402–405. 10.1016/j.appet.2009.12.013 20043964 PMC4372241

[40] Nowicka P, Sorjonen K, Pietrobelli A, Flodmark CE, Faith MS (2014) Parental feeding practices and associations with child weight status. Swedish validation of the Child Feeding Questionnaire finds parents of 4-year-olds less restrictive. Appetite 81: 232–241. 10.1016/j.appet.2014.06.027 24972134

[41] Geng G, Zhu Z, Suzuki K, Tanaka T, Ando D, Sato M, et al (2009) Confirmatory factor analysis of the Child Feeding Questionnaire (CFQ) in Japanese elementary school children. Appetite 52: 8–14. 10.1016/j.appet.2008.06.015 18657581

