# Supplementary material for: An FTO Gene Variant Moderates the Association between Parental Restriction and Child BMI
Source: PLoS One. 2016 May 19;11(5):e0155521. doi: 10.1371/journal.pone.0155521 (PMC4873182; doi:10.1371/journal.pone.0155521)
Supplement: S1 Table — (PDF) [file pone.0155521.s001.pdf]

Please read the following statements and circle what's most appropriate for **your child's** eating behavior.

1. Your child loves food.	Never / Rarely / Sometimes / Often /Always
2. Your child eats more when worried.	Never / Rarely / Sometimes / Often /Always
3. Your child has a big appetite.	Never / Rarely / Sometimes / Often /Always
4. Your child finishes his/her meal quickly.	Never / Rarely / Sometimes / Often /Always
5. Your child is interested in food.	Never / Rarely / Sometimes / Often /Always
6. Your child is always asking for a drink.	Never / Rarely / Sometimes / Often /Always
7. Your child refuses foods at first.	Never / Rarely / Sometimes / Often /Always
8. Your child eats slowly.	Never / Rarely / Sometimes / Often /Always
9. Your child eats less when angry.	Never / Rarely / Sometimes / Often /Always
10. Your child enjoys tasting new foods.	Never / Rarely / Sometimes / Often /Always
11. Your child eats less when he/she is tired.	Never / Rarely / Sometimes / Often /Always
12. Your child is always asking for food.	Never / Rarely / Sometimes / Often /Always
13. Your child eats more when annoyed.	Never / Rarely / Sometimes / Often /Always
14. If allowed to, your child would eat too much.	Never / Rarely / Sometimes / Often /Always
15. Your child eats more when anxious.	Never / Rarely / Sometimes / Often /Always
16. Your child enjoys a wide variety of foods.	Never / Rarely / Sometimes / Often /Always
17. Your child leaves food on his/her plate at the end of a meal.	Never / Rarely / Sometimes / Often /Always
18. Your child takes more than 30 minutes to finish a meal.	Never / Rarely / Sometimes / Often /Always
19. Given the choice, your child would eat most of the time.	Never / Rarely / Sometimes / Often /Always
20. Your child looks forward to mealtimes.	Never / Rarely / Sometimes / Often /Always
21. Your child gets full before his/her meal is finished.	Never / Rarely / Sometimes / Often /Always
22. Your child enjoys eating.	Never / Rarely / Sometimes / Often /Always

23. Your child eats more when he/she is happy.	Never / Rarely / Sometimes / Often /Always
24. Your child is difficult to please with meals.	Never / Rarely / Sometimes / Often /Always
25. Your child eats less when upset.	Never / Rarely / Sometimes / Often /Always
26. Your child gets full easily.	Never / Rarely / Sometimes / Often /Always
27. Your child eats more when he/she has nothing else to do.	Never / Rarely / Sometimes / Often /Always
28. Even if your child is full, he/she finds room to eat food he/she likes.	Never / Rarely / Sometimes / Often /Always
29. If given the chance, your child would drink continuously throughout the day.	Never / Rarely / Sometimes / Often /Always
30. Your child cannot eat a full meal if he/she has had a snack just before.	Never / Rarely / Sometimes / Often /Always
31. If given the chance, your child would always be having a drink.	Never / Rarely / Sometimes / Often /Always
32. Your child is interested in tasting food he/she hasn't tasted before	Never / Rarely / Sometimes / Often /Always
33. Your child decides that he/she doesn't like a food, even without tasting it.	Never / Rarely / Sometimes / Often /Always
34. If given the chance, your child would always have food in his/her mouth.	Never / Rarely / Sometimes / Often /Always
35. Your child eats more and more slowly during the course of a meal.	Never / Rarely / Sometimes / Often /Always
36. Your child 's stomach gets so full it hurts after eating.	Never / Rarely / Sometimes / Often /Always
37. Given the choice, your child would eat vegetables and fruits.	Never / Rarely / Sometimes / Often /Always
38. Given the choice, your child would drink sweet beverages (including fruit juices and drinks, soda, etc.) instead of water.	Never / Rarely / Sometimes / Often /Always

Please read the following statements and circle what's most appropriate for **you**.

39. My child chooses to eat healthy foods for snacks.	Never / Rarely / Sometimes / Often /Always
40. My child sits down together at a table with our family during mealtime.	Never / Rarely / Sometimes / Often /Always
41. My child eats snacks in front of the TV.	Never / Rarely / Sometimes / Often /Always
42. My child eats meals in front of the TV.	Never / Rarely / Sometimes / Often /Always
43. I encourage my child to eat more at mealtimes.	Never / Rarely / Sometimes / Often /Always

44. I encourage my child to finish what's on his/her plate.	Never / Rarely / Sometimes / Often /Always
45. I encourage my child to stop eating when he feels full.	Never / Rarely / Sometimes / Often /Always
46. I decide **what** my child eats.	Never / Rarely / Sometimes / Often /Always
47. I decide **how much** my child eats.	Never / Rarely / Sometimes / Often /Always
48. I decide **when** my child eats.	Never / Rarely / Sometimes / Often /Always
49. I decide **where** (which part of the house) my child eats.	Never / Rarely / Sometimes / Often /Always
50. I have to be sure that my child does not eat too many **sweets** (candy, ice cream, cake, cookies, etc.).	Disagree / Slightly Disagree / Neutral / Slightly Agree / Agree
51. I have to be sure that my child does not eat too many **snack foods** (potato chips, cheese puffs, popcorn, etc.).	Disagree / Slightly Disagree / Neutral / Slightly Agree / Agree
52. I have to be sure that my child does not eat too many **high-fat foods**.	Disagree / Slightly Disagree / Neutral / Slightly Agree / Agree
53. I have to be sure that my child does not eat too many **high-carb foods**.	Disagree / Slightly Disagree / Neutral / Slightly Agree / Agree
54. I have to be sure that my child does not eat too much of his **favorite foods**.	Disagree / Slightly Disagree / Neutral / Slightly Agree / Agree
55. I offer my child his favorite foods or sweets in exchange for good behavior.	Disagree / Slightly Disagree / Neutral / Slightly Agree / Agree
56. If I did not guide or regulate my child's eating, my child would eat too many **junk foods.**	Disagree / Slightly Disagree / Neutral / Slightly Agree / Agree
57. If I did not guide or regulate my child's eating, my child would eat too much of **his/her favorite foods**.	Disagree / Slightly Disagree / Neutral / Slightly Agree / Agree
58. I intentionally keep some foods out of my child's sight.	Disagree / Slightly Disagree / Neutral / Slightly Agree / Agree
59. I am concerned that my child is overweight.	Disagree / Slightly Disagree / Neutral / Slightly Agree / Agree
60. I am concerned that my child will become overweight.	Disagree / Slightly Disagree / Neutral / Slightly Agree / Agree

The following questions are general questions about your household.

61. What is your relationship to your child?
   a. Mother
   b. Father
   c. Step-mother
   d. Step-father
   e. Other ________________________

62. How would you describe your child relative to his/her siblings?
   a. No siblings, only child
   b. Youngest
   c. Middle
   d. Oldest

63. What is **your** marital status?
   a. Single, never Married
   b. Married
   c. Separated
   d. Divorced
   e. Widowed
   f. Other _____________________________

64. What ethnicity do you consider your child to be?
   a. Hispanic or Latino
   b. Not Hispanic or Latino
   c. Unknown

65. What race do you consider your child to be? Circle all that apply.
   a. American Indian or Alaska Native
   b. Asian
   c. Black or African American
   d. Native Hawaiian or Other Pacific Islander
   e. White
   f. Other ____________________________
   g. Unknown

66. What is the highest level of education that **you** have completed?
   a. Grade 8 or Less
   b. Some high school, no diploma
   c. High School graduate (Grades 9-12 or GED)
   d. Some post-high school, no degree
   e. Associates Degree
   f. College
   g. Professional School or Graduate School
   h. Other _____________________________

67. What is the highest level of education that **your spouse/partner** has completed?
   a. Grade 8 or Less

b. Some high school, no diploma
c. High School graduate (Grades 9-12 or GED)
d. Some post-high school, no degree
e. Associates Degree
f. College
g. Professional School or Graduate School
h. Other ____________________________
i. Not applicable

68. Do **you** rent or own your residence?
a. Rent
b. Own
c. Other

69. Including wages, salaries, self-employment, and any other sources of income, what was your total combined **household** income during the last 12 months?

a. Less than $25,000
b. $25,000-$65,000
c. $65,000-$145,000
d. $145,000-$225,000
e. Over $225,000
